# Supplementary material for: Anopheles mosquitoes reveal new principles of 3D genome organization in insects
Source: Nat Commun. 2022 Apr 12;13:1960. doi: 10.1038/s41467-022-29599-5 (PMC9005712; doi:10.1038/s41467-022-29599-5)
Supplement: Supplementary file 3 — Description of Additional Supplementary Files [file 41467_2022_29599_MOESM3_ESM.pdf]

**Description of Additional Supplementary Files**

File Name: Supplementary  Data 1
Description: Hi-C statistics

File Name: Supplementary Data 2
Description: PacBio sequencing statistics

File Name: Supplementary Data 3
Description: Long-distance chromatin loops detected using Hi-C method

File Name: Supplementary Data 4
Description: Pearson's correlation of compartments

File Name: Supplementary  Data 5
Description: List of genes within the loop anchors

File Name: Supplementary Data 6
Description: Polycomb loops

File Name: Supplementary Data 7
Description: 3D FISH statistics

File Name: Supplementary Data 8
Description: Percentage of repeats

File Name: Supplementary Data 9
Description: FISH probes
