# Supplementary material for: Anopheles mosquitoes reveal new principles of 3D genome organization in insects
Source: Nat Commun. 2022 Apr 12;13:1960. doi: 10.1038/s41467-022-29599-5 (PMC9005712; doi:10.1038/s41467-022-29599-5)
Supplement: Supplementary file 13 — Reporting Summary [file 41467_2022_29599_MOESM13_ESM.pdf]

nature portfolio

Corresponding author(s): Veniamin Fishman

Last updated by author(s): Mar 9, 2022

Reporting Summary

Nature Portfolio wishes to improve the reproducibility of the work that we publish. This form provides structure for consistency and transparency in reporting. For further information on Nature Portfolio policies, see our Editorial Policies and the Editorial Policy Checklist.

Statistics

For all statistical analyses, confirm that the following items are present in the figure legend, table legend, main text, or Methods section.

n/a	Confirmed	
☐	☒	The exact sample size (*n*) for each experimental group/condition, given as a discrete number and unit of measurement
☐	☒	A statement on whether measurements were taken from distinct samples or whether the same sample was measured repeatedly
☐	☒	The statistical test(s) used AND whether they are one- or two-sided<br>*Only common tests should be described solely by name; describe more complex techniques in the Methods section.*
☐	☒	A description of all covariates tested
☐	☒	A description of any assumptions or corrections, such as tests of normality and adjustment for multiple comparisons
☐	☒	A full description of the statistical parameters including central tendency (e.g. means) or other basic estimates (e.g. regression coefficient) AND variation (e.g. standard deviation) or associated estimates of uncertainty (e.g. confidence intervals)
☐	☒	For null hypothesis testing, the test statistic (e.g. *F*, *t*, *r*) with confidence intervals, effect sizes, degrees of freedom and *P* value noted<br>*Give P values as exact values whenever suitable.*
☐	☒	For Bayesian analysis, information on the choice of priors and Markov chain Monte Carlo settings
☐	☒	For hierarchical and complex designs, identification of the appropriate level for tests and full reporting of outcomes
☐	☒	Estimates of effect sizes (e.g. Cohen's *d*, Pearson's *r*), indicating how they were calculated

Our web collection on statistics for biologists contains articles on many of the points above.

Software and code

Policy information about availability of computer code

Data collection	No software was used for data collection.
Data analysis	Software used in the study:<br>Juicebox software (1.11.08);<br>3D-DNA pipeline (version 170123);<br>LastZ tool (https://doi.org/10.1016/j.brainres.2008.03.070);<br>KentUtils (https://github.com/ENCODE-DCC/kentUtils);<br>Mugsy (https://doi.org/10.1093/bioinformatics/btq665);<br>AQUAS ChIP-seq pipeline1 (https://github.com/kundajelab/chipseq_pipeline; https://www.ncbi.nlm.nih.gov/pmc/articles/PMC3431496/);<br>HISAT2 (Version 2.2.1);<br>deepTools (Version 3.3.2);<br>StringTie (Version 2.1.7);<br>Benchmarking Universal Single-Copy Orthologue (BUSCO v3.0.2);<br>MAFFT (v7.450);<br>AliStat (v1.12);<br>RAxML (v8.0.0, PROTGAMMAJTT model).<br><br>Source code for the ABCE tool computing cePC1 values is available here: https://github.com/labdevgen/ABCE.<br>Miscellaneous scripts are available at https://github.com/labdevgen/Anopheles_Ps; https://github.com/labdevgen/lavaburst_domains; https://github.com/labdevgen/ANopheles_Rabl.

For manuscripts utilizing custom algorithms or software that are central to the research but not yet described in published literature, software must be made available to editors and reviewers. We strongly encourage code deposition in a community repository (e.g. GitHub). See the Nature Portfolio guidelines for submitting code & software for further information.

Data

Policy information about availability of data

All manuscripts must include a data availability statement. This statement should provide the following information, where applicable:

- Accession codes, unique identifiers, or web links for publicly available datasets
- A description of any restrictions on data availability
- For clinical datasets or third party data, please ensure that the statement adheres to our policy

The raw sequencing data for five Anopheles species have been deposited in the NCBI SRA database with accession numbers PRJNA615788 (RNA-seq raw reads), PRJNA623252 (ChIP-seq raw reads), PRJNA615337 (Hi-C raw reads for embryos), and PRJNA630123 (Hi-C raw reads for An. merus adults). Processed data, including genome assemblies, Hi-C contact maps, RNA-seq and ChIP-seq tracks, TADs and compartments are available at https://genedev.bionet.nsc.ru/Anopheles.html. Genome assemblies are also available at NCBI (BioProject: PRJNA660041, genome accessions: JADFFJ000000000, JADGIR000000000, JADGIQ000000000, JADFFN000000000, JADFFO000000000). Note that the NCBI references are slightly different from those deposed at https://genedev.bionet.nsc.ru/Anopheles.html due to contamination filters applied by the NCBI team.

The data used for other organisms are publicly avialable and can be found under the following web links:
Silkmoth (Bombyx mori) (https://doi.org/10.1126/science.aal3327; https://doi.org/10.1101/254797; https://doi.org/10.1016/j.ibmb.2008.11.004);
Aedes aegypti (https://doi.org/10.1038/s41586-018-0692-z);
Drosophila melanogaster (embryo) (https://doi.org/10.1101/gad.328971.119);
Drosophila busckii (embryo) (https://doi.org/10.1101/gad.328971.119);
Drosophila viridis (embryo) (https://doi.org/10.1101/gad.328971.119) ;
Drosophila melanogaster (nc1-4, nc12, nc13, 3-4h embryo) (https://doi.org/10.1016/j.cell.2017.03.024);
Drosophila melanogaster Kc167 cells (https://doi.org/10.1073/pnas.1701291114; https://doi.org/10.1016/j.molcel.2017.07.022);
Drosophila melanogaster S2 cells (https://doi.org/10.1073/pnas.1901244116; https://doi.org/10.1038/s41467-017-02526-9);
Drosophila melanogaster BG3 (https://doi.org/10.1101/gr.246710.118);
Drosophila melanogaster salivary glands (Polytene) (https://doi.org/10.1016/j.cell.2015.10.026);
Drosophila melanogaster Rad21/CapH2 knockdowns (https://doi.org/10.1016/j.celrep.2019.01.116);
Chicken erythrocytes and fibroblasts (https://doi.org/10.1093/nar/gky1103);
DT40 at various cell cycle stages, including cells with condensin subunits depleted (https://doi.org/10.1126/science.aao6135);
Mouse neural progenitors, embryonic stem cells, mouse cortical neurons (https://doi.org/10.1016/j.cell.2017.09.043);
Mouse rod photoreceptors, thymus WT and LBR-/- cells (https://doi.org/10.1038/s41586-019-1275-3);
Human monocytes (https://doi.org/10.1016/j.molcel.2017.08.006);
Human HCT116 with/without RAD21 degron (https://doi.org/10.1016/j.cell.2017.09.026);
Human HAP1 with/without WAPL (https://doi.org/10.1016/j.cell.2017.04.013);
Human embryonic stem cells, human fibroblasts (https://doi.org/10.1016/j.molcel.2020.03.003).

Field-specific reporting

Please select the one below that is the best fit for your research. If you are not sure, read the appropriate sections before making your selection.

[x] Life sciences [ ] Behavioural & social sciences [ ] Ecological, evolutionary & environmental sciences

For a reference copy of the document with all sections, see nature.com/documents/nr-reporting-summary-flat.pdf

Life sciences study design

All studies must disclose on these points even when the disclosure is negative.

Sample size	The main parameter determining the amount of embryos used in Hi-C and ChIP-seq was a sufficient DNA quantity for experiment manipulations and for NGS-sequencing. We used 1000-3000 mosquito eggs per Hi-C/ChIP-seq/RNA-seq experiments. The exact number for each method is stated in the Methods section in manuscript text. The number of replicas was determined based on common standarts in genomic studies, such as minimum as 2 for Hi-C and ChIP-seq, 3 - for RNA-seq (ENCODE).
Data exclusions	No data were excluded from the analysis.
Replication	All Hi-C experiments were repeated at least in two replicas, ChIP-seq results were validated in 2 replicas, RNA-seq experiments were repeated in 3 replicas. All attempts at replication were successful and concordant for Hi-C, ChIP-seq, and RNA-seq experiments. The results of 2D and 3D FISH experiments were confirmed in several (>2) independent attempts.
Randomization	All samples were randomly allocated to experimental groups: all mosquito eggs were collected from the batch and fixed, without exceptions.
Blinding	The blinding parameter is not related to our study because we didn't have any a priori expectations (or hypothesis) regarding our results. For example, genome assembling is purely data-driven procedure which can not be influenced by operator's expectations.

Reporting for specific materials, systems and methods

We require information from authors about some types of materials, experimental systems and methods used in many studies. Here, indicate whether each material, system or method listed is relevant to your study. If you are not sure if a list item applies to your research, read the appropriate section before selecting a response.

Materials & experimental systems

n/a	Involved in the study
☐	☒ Antibodies
☒	☐ Eukaryotic cell lines
☒	☐ Palaeontology and archaeology
☐	☒ Animals and other organisms
☒	☐ Human research participants
☒	☐ Clinical data
☒	☐ Dual use research of concern

Methods

n/a	Involved in the study
☐	☒ ChIP-seq
☒	☐ Flow cytometry
☒	☐ MRI-based neuroimaging

Antibodies

Antibodies used	Anti-trimethyl-histone H3 (Lys27), Millipore, #07-449; dilution 1:100 for ChIP, 1:1000 for Western Blot
Validation	Before using the Anti-trimethyl-histone H3 (Lys27) antibodies for ChIP-seq, we have successfully validated them by Western Blot analysis. WB image is provided in Supplementary Materials (Supplementary Fig. 29).

Animals and other organisms

Policy information about studies involving animals; ARRIVE guidelines recommended for reporting animal research

Laboratory animals	All mosquito colonies were received from MR4 repository. The names of used strains are as follows: MOPTI strain (MRA-763), MAF strain (MRA-1156), EBRO strain (MRA-493), STECLA strain (MRA-126), Indian strain of Anopheles stephensi. We used embryonic material at developmental stage ~15-18 hours after oviposition. Embryos of mixed sexes were used.
Wild animals	The study didn't involve wild mosquitoes.
Field-collected samples	The study didn't involve samples collected from the field.
Ethics oversight	Virginia Polytechnic and State University (Virginia Tech), Fralin Life Science Institute

Note that full information on the approval of the study protocol must also be provided in the manuscript.

ChIP-seq

Data deposition

☒ Confirm that both raw and final processed data have been deposited in a public database such as GEO.

☒ Confirm that you have deposited or provided access to graph files (e.g. BED files) for the called peaks.

Data access links<br>*May remain private before publication.*	ChIP-seq raw reads: https://www.ncbi.nlm.nih.gov/sra/?term=PRJNA623252<br>ChIP-seq processed reads are available at https://genedev.bionet.nsc.ru/Anopheles.html
Files in database submission	ChIP-seq raw reads (repica 1, input 1, replica 2, input 2) for Anopheles atroparvus; AatrE3_V4.H3K27me3.fc.sifnal.bw (fold change file, replicas combined)
Genome browser session<br>(e.g. UCSC)	IGV, Juicebox tools

Methodology

Replicates	2
Sequencing depth	PE reads, 150 bp
Antibodies	Anti-trimethyl-histone H3 (Lys27), Millipore, #07-449
Peak calling parameters	Aquas pipeline (https://github.com/kundajelab/chipseq_pipeline) stopped at the signal stage.
Data quality	Western Blot validation; correspondence with A/B compartments

Software	Aquas pipeline (https://github.com/kundajelab/chipseq_pipeline) stopped at the signal stage
